# MiR-200a Regulates Nasopharyngeal Carcinoma Cell Migration and Invasion by Targeting MYH10

**DOI:** 10.7150/jca.40438

**Published:** 2020-03-04

**Authors:** Wenya Liu, Tonghui Cai, Lingjun Li, Hui Chen, Ruichao Chen, Minfen Zhang, Wei Zhang, Li Zhao, Hanzhen Xiong, Ping Qin, Xingcheng Gao, Qingping Jiang

**Affiliations:** 1 Department of Pathology, the Third Affiliated Hospital, Guangzhou Medical University, Guangzhou, China 510150; 2 Department of Pathology, the First Affiliated Hospital, Anhui Medical University, Hefei, China 230022; 3 The First Affiliated Hospital, Guangzhou Medical University, Guangzhou, China 511436

**Keywords:** nasopharyngeal carcinoma, MYH10, miR-200a

## Abstract

Nasopharyngeal carcinoma (NPC), is one of the most common malignant tumor in southern China and southeast Asia. MYH10 is a coding gene of the NMMHC-IIB protein. Previous studies have shown that MYH10 expression was up-regulated in breast cancer, glioma and meningioma. Moreover, it was targeted by miR200 family. However, no relevant studies have been found in NPC. In present study, we found in 48 NPC specimens, MYH10 level was lower in most cancer areas than that in the adjacent normal tissue. Moreover, the depletion of MYH10 can promote the migration and invasion of NPC. In addition, we demonstrated that miR-200a has the strongest regulation to MYH10 among miR-200 family. miR-200a mimics could decrease MYH10 expression, while miR-200a inhibitor increase MYH10 expression. Next, we found that miR-200a bound directly to MYH10 using Dual-luciferase reporter. Finally, it was demonstrated that siMYH10 could reverse the effect of miR-200a inhibitor on NPC cell migration and invasion. Taken together, it can be concluded that MYH10 is lowly expressed in NPC compared with adjacent tissues, and the loss of MYH10 can promote the migration and invasion of NPC cells; Among the miR-200 family, miR-200a has the strongest regulatory effect on MYH10; MYH10 is a direct target gene of miR200a, and miR200a targets MYH10 to regulate the migration and invasion of NPC cells.

## Introduction

Nasopharyngeal carcinoma (NPC) is one of the most common malignant tumors of the head and neck. It mainly occurs in the top and lateral walls of the nasopharyngeal cavity and is associated with heredity, environment and Epstein-Barr virus (EBV) infection. NPC is rare in the world, but particularly high in north Africa, southeast Asia and east Asia, especially in Guangdong China^1^. Nasopharyngeal carcinoma is a highly metastatic and invasive malignant tumor which the lymph node metastasis can occur in the early stage, and most patients cannot be cured in advance^2^.

MYH10 is located on human chromosome 17 which encoding protein Non-muscle myosin II (NMII). As the main regulator of cell morphology, NMII plays a key role in many basic cell processes, including vesicle transfer and repair, cell migration, cytokinesis, and maintenance of cell polarity. Current tumor studies related to MYH10 have shown that MYH10 plays different roles in different tumors. It has been reported that mosaic loss of NMIIA and NMIIB can lead to mammary epithelial proliferation. According to previous studies, MYH10 may cause cells to move in the opposite direction, and tumor suppressor p53 can reduce RhoA activation and change NMIIB expression, both of which can simultaneously reduce tumor invasion 3-4. At present, the role of MYH10 in tumors is rarely reported, and the influence of MYH10 on the occurrence and development of nasopharyngeal carcinoma is an area that has not been studied yet.

In the study about meningiomas, miRNA-200 regulates the development of tumor through binding MYH10. The miR-200 family, composed of miR-200a, miR-200b, miR-200c, miR-141 and miR-429, mainly inhibits EMT by targeting the 3 'UTRS of ZEB1 and ZEB2 to inhibit tumor invasion and metastasis 5, which is considered as a tumor suppressor miRNA. miR200 is low- expressed in lung cancer 6, oral squamous cell carcinoma 7, liver cancer 8, moreover, low expression of miR200 in ovarian cancer is associated with poor prognosis 9, the overexpression of miR-200 can reduce the invasion and metastasis of tumor cells 10-13. However, miR-200b has been reported to promote cell proliferation and invasion of T-cell acute lymphoblastic leukemia 14. Our previous study showed that miR200c can promote the invasion and migration in endometrioid endometrial carcinoma. 15. Interestingly, miR200 has been reported to regulate the development of meningiomas by combining MYH10, but there has been no relevant study on this regulatory effect in nasopharyngeal carcinoma. Therefore, our research group will investigate the effect of miR-200 family and MYH10 on NPC.

## Materials and Methods

### Patients and clinical samples

48 cases of human NPC paraffin specimens including cancer and matched adjacent normal tissues were obtained from Third Affiliated Hospital of Guangzhou Medical University from 2013 to 2018. All the patients provided informed consent, and the study was approved by the ethics committee of Third Affiliated Hospital of Guangzhou Medical University.

### Immunohistochemistry staining (IHC)

Using Immunohistochemistry to detect the MYH10 (Abcam, UK, 1:100)protein expression in the 48 NPC samples. All specimens were fixed in 10% neutral formalin, routinely dehydrated, embedded in paraffin, and serially sectioned at 4 μm thick. After DAB staining, counterstained the slices with hematoxylin, and mounted in neutral gum to observe the paraffin sections. The tissues were then analyzed by a bright-field microscope. Positive reaction was defined as showing brown signals in the cell cytoplasm. For MYH10 a staining index (0-10) was determined by multiplying the score for staining intensity with score for positive area. The expression of MYH10 was respectively defined as negative (0), weak positive (1), medium positive (2) and strong positive (3).The frequency of positive cells was defined as follow:0, less than 5%; 1, 5%-25%,; 2, 26%-50%; 3, 51%-75%; 4, greater than 75%.

### Cell lines and culture conditions

Human nasopharyngeal carcinoma cell lines 5-8F, HONE1-EBV, SUNE1 and CNE2 were the gift of the Basic College of Guangzhou Medical University. HONE1 was purchased from Jennio biotechnology company in Guangzhou.All of the cell lines were cultured in RPMI 1640 (Corning, USA) supplemented with 10% fetal bovine serum (FBS; Gibco, USA) and 50µg/ml of penicillin and streptomycin under the condition of 37 ºC and 5% CO2.

### Cell transfections

siMYH10 and the Negative Control was designed and synthesized by Guangzhou Ribobio company, cells were plated into six-well plates (1×10 6 cell/well) and transfected with siMYH10 using riboFECT (Ribobio, Guangzhou, China) according to the manufacturer's protocol.The miR-200 family mimic and miR-200a inhibitor primer, which were synthesized by Ribobio(Guangzhou, China), was transfected to NPC cells in the same way.

### RNA extraction and quantitative real-time PCR (qRT-PCR)

Total RNA was isolated from cells by using TRIzol™ Reagent (Invitrogen,USA). After reverse transcription into cDNA with Reverse Transcription Kit (Takara, Japan)and subjected to qRT-PCR using TB Green®Premix Ex Taq™(Takara, Japan) according to the manufacturer's instructions. The miR-200 family specific primers and U6 were synthesized by Ribobio (Guangzhou, China). QRT-PCR cycling conditions were 95°C for 30s, 40 cycles at 95°C for 5s, 60°C for 30s. Beta-actin acts as an internal reference in MYH10 while U6 plays the same role in miRNA. The qRT-PCR reactions were performed using an ABI Step One Plus instrument (Applied Biosystems, Foster City, CA). All the primer sequences used are listed in Supplementary Table 1.

### Western blot analysis

Cells were lysed using RIPA lysis buffer (Biosharp, China) containing 1%PMSF. Total protein samples were separated by 8% SDS-PAGE and then electro-transferred to PVDF membranes (Millipore, USA). After blocking with 5% BSA in TBST for 2h at room temperature, the PVDF membrane was probed with the MYH10 primary antibody (Abcam, UK, 1:1000) and anti-GAPDH antibody (Proteintech, USA, 1:1000) at 4℃ overnight. Afterward, membranes were incubated for 2h with a secondary antibody (Proteintech, USA, 1:5000 dilution). Signal was observed using Pierce® ECL Western Blotting Substrate (Thermo,USA), and the proteins were detected and quantified by ChemiDoc-XRS+(Bio-Rad, CA).

### Dual-luciferase reporter assay

The sequence of MYH10 in the pcDNA3.1 plasmids were designed and amplified by PCR and sub-cloned into the psiCheck2.0 vector to luciferase reporter assay. The resulting constructs were named psi-MYH10-WT, psi-MYH10-Mut. Briefly, 293T cells were incubated in 24-well plates and co-transfected with 100 ng of psi-check 2.0 luciferase vectors containing the MYH10 WT or Mut, and miR-200a mimics/inhibitor or NC according to the experimental groups. The Dual-Luciferase Reporter Assay (Promega, CA) was performed according to the manufacturer's instructions. All the transfection experiments were performed in triplicate.

### Cell migration and invasion assay

Migration and invasion assays were performed in duplicate using migration chambers (8-mm pore size,Costar) with Matrigel (BD Biosciences). NPC cell line was transfected for 24h before seeded into the upper chambers of transwells with serum-free medium, while the lower chambers filled with medium containing 10% FBS medium, The cells were placed in incubator for several hours, then fixed in methanol for 1 hour or overnight before stained with 0.1% crystal violet for 30min. Cells on the lower surface were photographed, and counted five random fields of cells.

### Wound healing assay

Evenly draw least 3 cross lines with marker pen about every 1cm at the back of the 6-well plate. One day before transfection, equal numbers of NPC cells (6X10⁶) were seeded into 6-well plates. Cells were then transfected with 50nM siMYH10 using riboFECT (Ribobio, Guangzhou, China). At around 24h post-transfection, a wound was made perpendicular to the draw lines on cell layer with the use of a sterile plastic 200ul micropipette tip. After wounding, cells were cultured with 2% FBS medium. At different time points, cells that migrated into the wounded area or cells with extended protrusion from the border of wound were photographed under an inverted microscope.

### Statistical analyses

GraphPad Prism 7.0 software was used to analyze and process the experimental data. T test was used to analyze and compare between two groups, *P*<0.05 was considered statistically significant (*). Data were expressed as the mean ± standard deviation of at least three separate experiments.

## Result

### Expression of MYH10 in NPC tissues and cell lines

**Figure 1:**
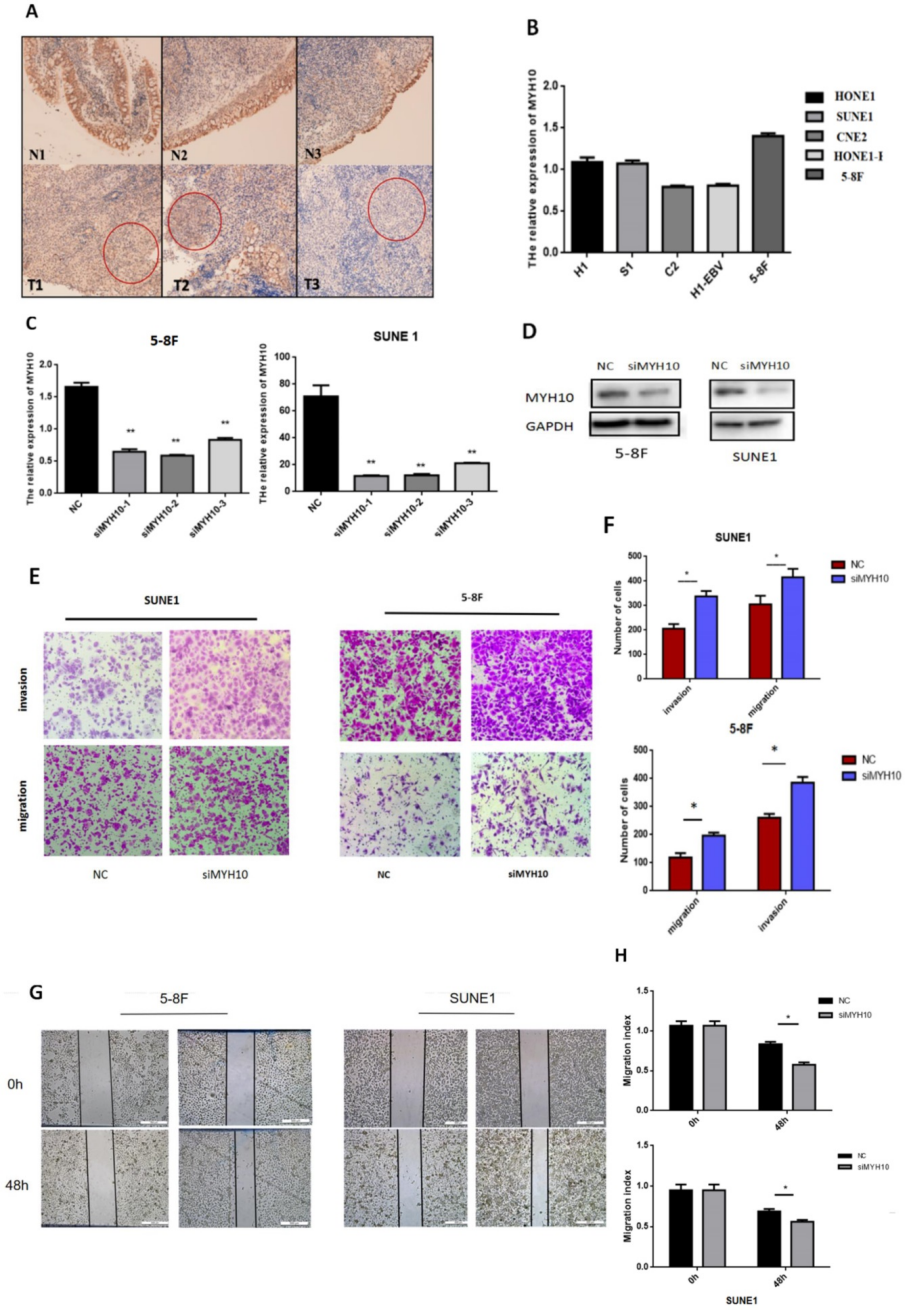
** Inhibition of MYH10 expression increased the migration and invasion of NPC cells. A.** Immunohistochemistry showed that the expression of MYH10 in adjacent normal tissues is stronger than nasopharyngeal carcinoma tissues. **B.** In five NPC cell lines the expression of MYH10 was detected by qRT-PCR.** C.** After transfected with three groups of siMYH10, qRT-PCR dected the mRNA level of MYH10.** D.** Western blot showed that the expression of MYH10 protein decreased after transfected with siMYH10.** E,F.** Migration and invasion assay in 5-8F and SUNE1 NPC cells that were transfected with siMYH10 for 48h. Cells were evaluated at 12 h(migration)/24h(invasion) after transfection (×200 magnifification). **G,H.**Wound healing assay showed that NPC cells transfected with siMYH10 for 24h, pictures were taken by 48h after wounding.The results are shown as the mean ± SEM from three independent experiments (*P < 0.05).

Immunohistochemistry was used to detect the expression of MYH10 in 48 cases of nasopharyngeal carcinoma matched samples. The results found that, compared with the normal tissues, MYH10 expression was lower in NPC tissues (77.08%, 37/48). In 2 cases, MYH10 index was higher than normal tissues, accounting for 4.17% In other 9 samples, the positive index of cancer tissue and normal tissue was equal, accounting for 18.75% (Fig. 1A).

The expression level of MYH10 in different nasopharyngeal carcinoma cell lines was detected by qRT-PCR, and the results demonstrated that the expression level of MYH10 in 5-8F was the highest, and the expression level in CNE2 and HONE1-EBV cells was low (Fig. 1B).

The 5-8F and SUNE1 cell lines with the highest expression level of MYH10 were selected as the experimental cells. Three groups of MYH10 siRNA were used to inhibit the expression of MYH10 in 5-8F and SUNE1 cells respectively. After transfection of siMYH10 for 48h, the expression of MYH10 was detected by qRT-PCR and western blot (Fig. 1C,D).

### Inhibiting the expression level of MYH10 in nasopharyngeal carcinoma cell lines can enhance the ability of cell migration and invasion

When inhibiting the expression of MYH10, the ability of cell migration and invasion was enhanced by transwell assay (Fig. 1E). Furthermore, the wound healing assay was also used to detect the effect of MYH10 on cell migration, and the healing ability of NPC cells increased with the low expression of MYH10 (Fig. 1G). The results showed that knockdown of MYH10 could lead to stronger invasion and migration in NPC cells (*P* < 0.05, Fig. 1F,H).

### The miR200 family regulates the expression of MYH10

**Figure 2:**
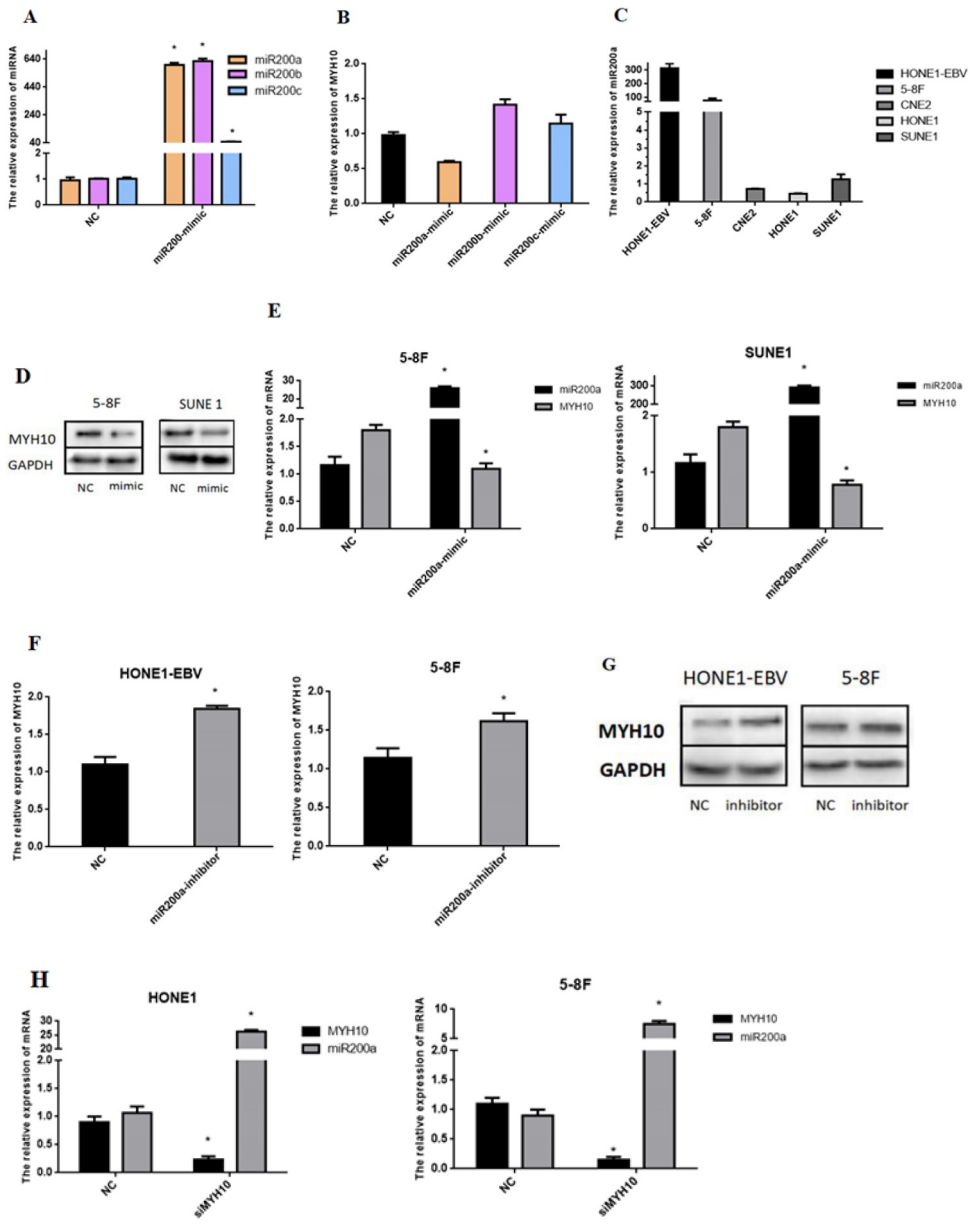
** The relationship between miR-200a and MYH10. A,B.** MYH10 was regulated by transfecting NPC cells with miR-200-mimics, respectively. **C.** miR-200a was detected by qRT-PCR in five NPC cells line. **D-G.** qRT-PCR and Western-blot demonstrated that the expression of MYH10 were decreased and increased after transfected with miR-200a-mimic/miR-200a-inhibitor by 48h in NPC cells. **H.** miR-200a was upregulated in HONE1 and 5-8F cell lines when MYH10 expression was inhibited. All results are presented as the mean of triplicate assays ( *P < 0.05).

To investigate the regulatory effect of miR-200 family on MYH10, we transfered miR-200a, miR-200b and miR-200c mimics of miR-200 family into NPC cell lines, respectively. qRT-PCR was used to detect the changes in the expression level of MYH10 after overexpression of miR-200 family. However, MYH10 expression was decreased by about 1 times after transfected with miR-200a-mimics, but increased about 0.5 times after overexpression of miR-200b, and after overexpression of miR200c, MYH10 slightly increased (Fig. 2A,B). Therefore, miR-200a with the strongest regulation in MYH10 was selected to continue the following experiment. qRT-PCR was used to detect the expression level of miR-200a in different nasopharyngeal cancer cell lines. The results showed that miR-200a was most highly expressed in HONE1-EBV cell line, followed by high-metastatic cell line 5-8F, with low expression in SUNE1, CNE2 and HONE1 cell lines (Fig.2C).

### MYH10 is a target gene of miR-200a

To further investigate the relationship between miR-200a and MYH10 in NPC we transfected the miR-200a-mimic or miR-200a-inhibitor into different NPC cells using transient transfection, and observed the changes in the expression levels of miR-200a and MYH10. The expressions of miR-200a and MYH10 were detected by qRT-PCR (Fig. 2E,F). Data showed that compared with the NC control group, the expression level of MYH10 after transfection with miR-200a-mimic in 5-8F and SUNE1 was down-regulated (*P*<0.05), and the expression level of MYH10 was up-regulated after transfection with miR-200a-inhibitor in HONE1-EBV and 5-8F (*P*<0.05). Western blot assay demonstrated the same outcome. The results showed that up-regulation or down-regulation of miR-200a could inhibit or increase the expression of MYH10 in NPC cells, miR-200a was negatively correlated with MYH10 (Fig. 2D,H).On the contrary, after interfering with the expression of MYH10, qRT-PCR was used to detect the expression level of miR-200a in NPC cells (Fig. 2H). We found that the miR-200a expression level was increased after transfected with siMYH10, further proving that MYH10 and miR-200a could be mutually regulated.

### MYH10 is a direct target of miR-200a

**Figure 3:**
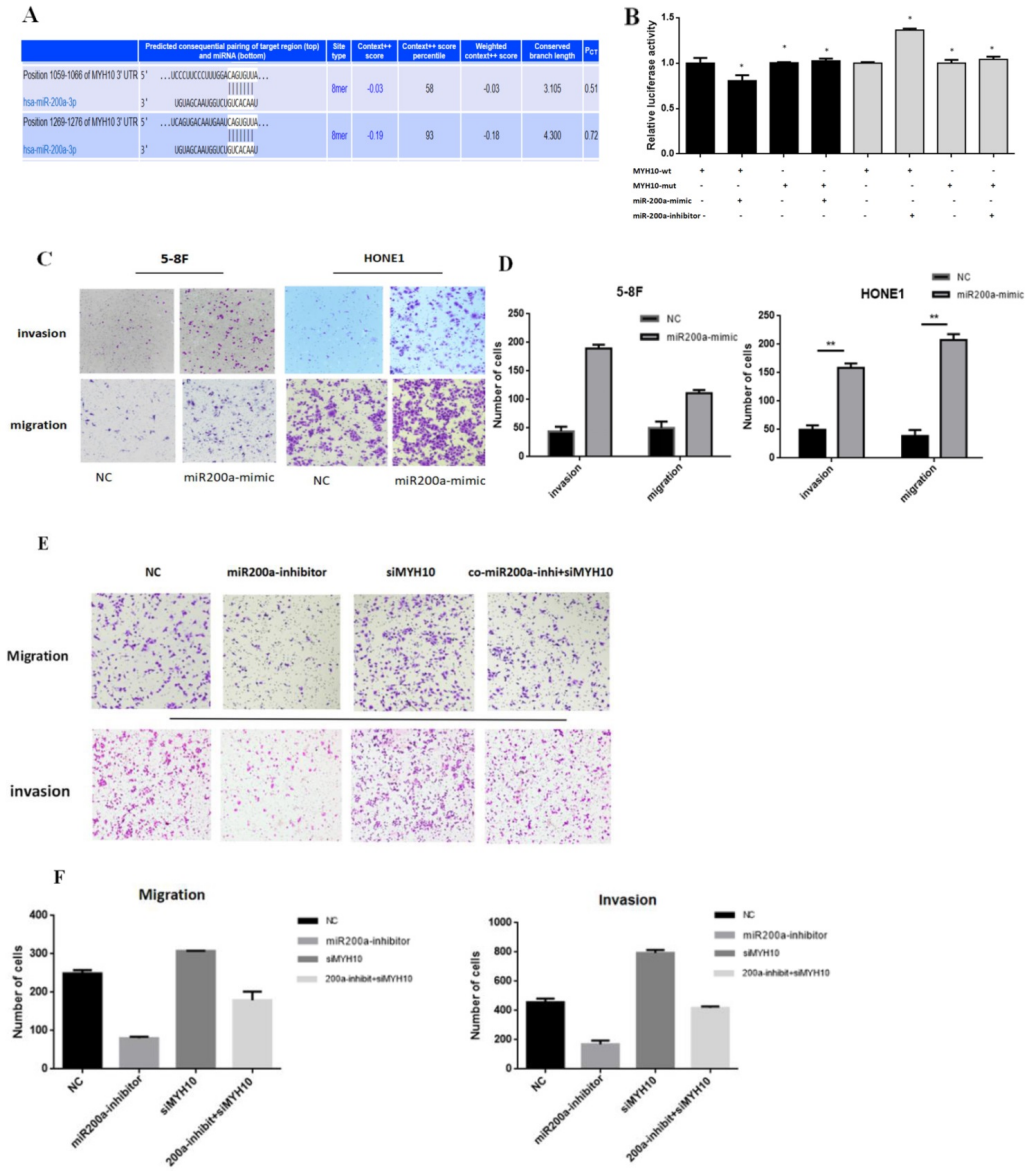
** miR-200a target MYH10 directly. A.** Targetscan predicted that MYH10 have bound to miR-200a directly. **B.** Dual-luciferase reporter by transfecting 293T cells with miR-200a-mimics and miR-200a-inhibitor to verify the interaction between the MYH10 and miR-200a.** C, D.** Migration and invasion assay in 5-8F and HONE1 NPC cells that were transfected with miR-200a-mimic for 48h. Cells were evaluated at 12 h/24h after transfection (×200 magnification). **E,F.** Transfected NC/miR-200a inhibitor/siMYH10/co miR-200a-inhibitor and siMYH10 into HONE1 EBV cell for migration and invasion assay, evaluated the results after 12h/24h. All results are presented as the mean of triplicate assays.( *P < 0.05).

According to the research results, we speculated that miR-200a may exist at the binding sites with MYH10. TargetScan wedsite was used to predict their combination, the details of which were presented in Fig 3A, which showed that in the MYH10 promoter region there was base paired seed area with miR-200a. To further study the binding mechanism between miR-200a and MYH10, we constructed wild-type (WT) and mutant (Mut) luciferase reporter vectors according to the binding sites. The results showed that upregulation of miR-200a expression level could cause the decrease of luciferase activity of MYH10, but the phenomenon disappeared after the mutation of binding site. The dual-luciferase reporter proved that MYH10 was the target gene of miR-200a (Fig.3B).

### Effects of miR-200a on invasion and migration of NPC cells and rescue experiments

The HONE 1(with the lowest miR-200a expression) and 5-8F (with high expression of MYH10) cell lines were transfected into a miR-200a-mimic. The results of the Transwell showed that the ability of migration and invasion in NPC cell increased after miR-200a was overexpressed (Fig. 3C,D). In another experiment, HONE1 EBV cell line was divided into four groups. The negative control group was set up in the first group; in the second group, miR-200a expression was inhibited; in the third group, siMYH10 was transfected to interfere with MYH10 expression; in the fourth group, miR-200a-inhibitor and siMYH10 were co-transfected to observe whether reducing the expression of MYH10 could save the effect of miR-200a-inhibitor on cell migration and invasion. It showed that after the inhibition of miR-200a expression, the invasion and migration ability of NPC cell was decreased; and the invasion and migration ability of NPC cell was enhanced when MYH10 was inhibited; the inhibitory effect of miR-200a-inhibitor decreased, after MYH10 was interfered. It suggested that co-transfection of siMYH10 could partially reverse the inhibitory effect of miR-200a-inhibitor on the invasion and migration ability of NPC.

After transfected with siMYH10, miR-200a-inhibitor can partially save the effect of siMYH10 on promoting the invasion and migration of NPC (Fig. 3E,F).

## Discussion

NPC is a type of squamous cell carcinoma derived from malignant tumour of the head and neck. The etiology mainly includes virus (EBV) infection, ethnicity, locality, genetic susceptibility, environmental factors and dietary factors 16. Compared with other head and neck tumors, NPC is more likely to metastasize in the late stage.

MYH10 gene is located on human chromosome 17. Non-muscular myosin IIB (NM IIB) is a protein encoded by MYH10 gene. It belongs to myosin superfamily and is involved in tumor cell migration, invasion, extracellular matrix (ECM) production and epithelial-mesenchymal transformation (EMT). Betapudi V et al. 17 have reported that MYH10 gene is overexpressed in breast cancer, and MYH10 is associated with tumor cell invasion in breast cancer. However, other studies have shown that mosaic loss of MYH9 and MYH10 can lead to excessive proliferation of breast epithelial cells 18. In the latest studies on glioma, inhibition of MYH10 expression can reduce the ability of glioma cells to migrate and invade 19.

In our present study, among 48 pairs of nasopharyngeal carcinoma paraffin specimens, in 77.08% of the normal samples MYH10 is stronger than in the samples of cancer tissues. In the relevant cell function experiments, we found that the invasion and migration ability of nasopharyngeal carcinoma cells were enhanced after the MYH10 was reduced *in vitro*. These experimental results all indicated that MYH10 had an anti-cancer effect.

According to previous reports, MYH10 tends to act as an oncogene in tumors, and this study found for the first time that MYH10 has low expression in nasopharyngeal carcinoma. As we known, NMII A (MYH9) and NMII B (MYH10) both belong to the NMII family, which is normally expressed in tumor 20. However, MYH9 also exists as a tumor suppressor. Schramek et al. firstlty found that silencing MYH9 induced metastatic SCCs in the skin and head and neck. In the cutin cells of mice or humans, MYH9 can regulate the post-transcriptional stabilization of P53. The authors found that 24-31% of human skin and head and neck SCCs are characterized by no or very weak NMIIA expression 21. Interestingly, another mouse model, in which Myh9 was ablated in the heart and the tongue epithelium, showed the development of tongue SCC 22. In our series of clinical specimen verification and *in vitro* cell experiments, MYH10 has been confirmed to play a consistent role with MYH9 as a tumor suppressor gene in nasopharyngeal carcinoma. We hypothesized that the inhibitory effect of MYH10 in nasopharyngeal carcinoma was related to the co-polymerization of NMIIA (MYH9) and NMIIB (MYH10) 23-24. When MYH10 expression is externally inhibited, the decrease of NMIIB protein reduces the binding NMIIA protein at the same time, leading to instability of P53, which increased migration and invasion of NPC cells. In addition, many studies have shown that malignant transformation is usually accompanied by the destruction of the cytoskeleton, MYH10 can maintain the integrity and stability of the cytoskeleton. Furthermore, recovering cytoskeletal network can reverse transformation phenotype, namely, cell shape, cell adhesion, the density of saturated, this may also be the anticancer mechanism of MYH10 25-28. Finally, Xia M. et al. reported that MYH10 can make cells move in the opposite direction, and the tumor suppressor P53 can reduce RhoA activation and change MYH10 expression in mice^29^-^30^. In conclusion, the tumor inhibition mechanism of NMII family maybe closely related to P53. That's what we are working on.

MYH10 has been rarely reported in tumors. In meningioma, overexpression of miR-200a can down-regulate the expression of MYH10, leading reduction of the migration and invasion in meningioma cells 31. In meningiomas, miR-200a has been shown to inhibit the metastasis and invasion of meningiomas by directly binding 3 'UTRS of MYH10. In lung cancer, the downregulation of BMP4 caused by overexpression of miR-200a can inhibit the development and migration ability of tumor cells. MYH10, as a downstream gene of BMP4, may be indirectly regulated by miR200a 32. Therefore, we assume that miR-200a may regulate MYH10 expression through these two pathways.

In present study, we discovered that miR-200a had the strongest regulatory effect on MYH10 among miR-200 family. MYH10 was down-regulated after NPC cell lines transfection with miR-200a-mimic and up-regulated with miR-200a-inhibitor. After inhibiting MYH10 expression with siMYH10, qRT-PCR detected a significant increase in miR-200a, which may be related to the mutual regulation between miR-200a and MYH10. Both results indicate that miR-200a and MYH10 can bind each other. We then verified miR-200a and MYH10 can combine directly by bioinformatics analysis and Dual-luciferase reporter assay. In cell function experiments, inhibition of miR-200a can partially reverse the effect of MYH10 on migration and invasion of nasopharyngeal carcinoma cells. In our study, we found that overexpression of miR-200a can promote the migration and invasion of 5-8F and HONE1 cell lines of nasopharyngeal carcinoma. Shi, Z. et al. found that in nasopharyngeal carcinoma, the nuclear factor-κB (NF-κB) signaling pathway was highly active. In this report, miR-200a was shown to enhance the proliferation of nasopharyngeal carcinoma cells by activating the NF-κB signaling pathway, thereby exerting the role of oncogene 33. The role of miR-200a in nasopharyngeal carcinoma is still debated, it may be associated with cell line selection, but in our subsequent study, we found that in 5-8F, SUNE1, HONE1 cell lines, miR-200a can promote the migration of the nasopharyngeal carcinoma cell, but miR-200a expression or reduce has no effect on cell proliferation and expression. In cell function experiments, after co-transfection with miR-200a-inhibitor and siMYH10, it was found that the effect of inhibiting miR-200a on cell invasion and migration was reversed after MYH10 knockdown. Therefore, we believe that MYH10 is an important pathway for miR-200a to regulate nasopharyngeal carcinoma cell migration and invasion.

## Supplementary Material

Supplementary table.
